# Supplementary material for: ERRATUM: Prevalence and characteristics of lesions in elderly people living in the community
Source: Rev Esc Enferm USP. 2024 Sep 27;58:e2024ER08. doi: 10.1590/1980-220X-REEUSP-2024-ER08en (PMC11441791; doi:10.1590/1980-220X-REEUSP-2024-ER08en)
Supplement: Translation

# Translation: ERRATA: “Prevalência e características das feridas em pessoas idosas residentes na comunidade”

No artigo “Prevalência e características das feridas em pessoas idosas residentes na comunidade”, com número de: DOI: https://doi.org/10.1590/S0080-623420150000700008, publicado na Revista da Escola de Enfermagem da USP, volume 49 (Esp) de 2015, p. 51–57, na página 57:

**Leia-se:**

## Apoio financeiro

O presente trabalho foi realizado com apoio da Coordenação e Aperfeiçoamento de Pessoal de Nível Superior – Brasil (CAPES) – Código de Financiamento 001.
